# The association between eosinophilic exacerbation and eosinophilic levels in stable COPD

**DOI:** 10.1186/s12890-021-01443-4

**Published:** 2021-03-02

**Authors:** Hye Seon Kang, Sung Kyoung Kim, Yong Hyun Kim, Jin Woo Kim, Sang Haak Lee, Hyung Kyu Yoon, Chin Kook Rhee

**Affiliations:** Division of Pulmonology, Allergy and Critical Care Medicine, Department of Internal Medicine, Seoul St. Mary’s Hospital, College of Medicine, The Catholic University of Korea, 222 Banpo-daero, Seocho-gu, Seoul, 06591 Republic of Korea

**Keywords:** Blood, Eosinophilia, Exacerbation

## Abstract

**Background:**

Blood eosinophil count may predict treatment response in patients with chronic obstructive pulmonary disease (COPD) during acute exacerbations (AE). However, the ability and thresholds of blood eosinophil counts in stable status to predict eosinophilic AECOPD have not been completely investigated.

**Methods:**

This was a retrospective multicenter study performed January 2010 to December 2014. COPD subjects hospitalized with exacerbations, were included. Blood samples were obtained at the time of AE and stable disease at outpatient clinic before or after admission. We identified a blood eosinophil count cut-off point at stable COPD, either taken as a percentage or as absolute value, for identification of eosinophilic exacerbation.

**Results:**

There was significant positive correlation of eosinophil counts between stable COPD and AECOPD. The best cut-off value of blood eosinophil count in stable status for the prediction of eosinophilic COPD exacerbation based on blood eosinophil count ≥ 2% was 300 cells/µL (area under the ROC curve [AUC] 0.614, *P* = 0.001, 39% sensitivity, 83.8% specificity). When the eosinophilic COPD exacerbation was based on blood eosinophil count ≥ 300 cells/µL, the best cut-off value of blood eosinophil count in stable status for the prediction of eosinophilic COPD exacerbation was also 300 cells/uL (AUC 0.634, *P* = 0.046, 45.8% sensitivity, 80.9% specificity).

**Conclusions:**

We demonstrated association between blood eosinophil counts at stable COPD and those with AECOPD. The thresholds of blood counts at stable COPD to predict eosinophilic exacerbations was 300 cells/µL. Further and prospective studies in other populations should validate our results.

## Background

Chronic obstructive pulmonary disease (COPD) is a heterogenous disease. Phenotype-specific biomarkers to direct therapy were investigated [1]. Peripheral blood eosinophilia has been suggested as an one of useful marker of sputum eosinophilia during acute exacerbation (AE) COPD and stable COPD even though the association between blood and sputum eosinophilia still have controversies [2–5]. The study by Kitagushi et al*.* showed that increased steroid responsiveness was observed in COPD patients with asthma [6]. Eosinophilic exacerbations experienced better clinical outcomes than did those with neutrophilic exacerbations in COPD patients [7, 8]. However, few studies on the association between eosinophil counts in AECOPD and stable COPD exist, and the ability and thresholds of blood eosinophil counts to predict eosinophilic COPD exacerbations has not been completely investigated.

There is controversy regarding the use of blood eosinophil levels as biomarkers of exacerbation risk because of significant variability throughout the course of COPD [9]. Blood eosinophils at a time-point were a useful predictor of being in the persistent eosinophilia group over the next 12 months demonstrating longitudinal stability of blood eosinophilic inflammation within individuals. Eosinophilic inflammation groups based on blood eosinophils ≥ 2% had higher eosinophilic exacerbation rates than intermittent eosinophilic or rarely eosinophilic groups in COPD [10].

Eosinophilic COPD is distinct phenotype of the disease, and prediction of eosinophilic AECOPD is integral. However, little is known about the association between eosinophil counts in stable COPD and AECOPD. In this study, we compared the clinical outcomes in AECOPD patients with and without eosinophilia. We investigated if blood eosinophil counts in stable COPD and AECOPD are associated. Also, we stratified patients by their percentage and absolute number of blood eosinophils at stable COPD to investigate thresholds of eosinophil counts to predict eosinophilic AECOPD.

## Methods

This was a multicenter retrospective study performed between January 2010 and December 2014 in six university affiliated hospitals in the Republic of Korea. This study was approved by the Clinical Research Ethics Committee of the Catholic Medical Center (approval number: XC16RIMI0030). All data were collected from hospital databases. The requirement for informed consent was waived by the boards because the study was based on retrospective medical chart reviews [7].

### Patients

COPD subjects age older than 40, post-bronchodilator forced expiratory volume in 1 s (FEV_1_) and forced vital capacity (FVC) ratio < 0.7, hospitalized with exacerbations, were included. Patients with underlying lung cancer, which is identified by chest CT at the time of hospital admission due to AECOPD or patient history taking; who chronically used steroids in the case of steroid dependent patients; who were admitted because of other medical problems and who exhibited definite pneumonic infiltrations on chest X-ray at the time of admission, were excluded. Stable COPD was defined as no use antibiotics or oral corticosteroids, no increase in bronchodilator use, no unscheduled doctor’s visit, or no hospitalization due to COPD AE in the past 4 weeks [3]. We defined eosinophilic exacerbations as a peripheral blood eosinophil count ≥ 300 cells/μL and/or ≥ 2% of the total leukocyte count.

### Data

We extracted the following data from the medical records: patients’ demographics; history of smoking; the number of hospital or emergency room admissions in the previous year; the types of regular COPD medications taken; laboratory data (eosinophil counts during stable COPD and AECOPD at the time of hospital admission); PFT results (at stable COPD); hospital days; admission to the intensive care unit (ICU); length of ICU stay; any need for mechanical ventilation (MV); the duration of MV; any need for non-invasive ventilation; and treatment outcomes.

Blood samples were obtained at the time of AEs (hospital admission due to AECOPD) and stable disease at outpatient clinic before or after admission. Blood eosinophils were measured during the automated full blood count analysis. By constructing receiver operating characteristic (ROC) curves, we identified a blood eosinophil count cut-off point at stable COPD, either taken as a percentage or as absolute value, for identification of predicting blood eosinophil count at AECOPD.

### Statistical analysis

Baseline demographics and clinical outcomes were compared between patients with eosinophilia and non-eosinophilia. We used Pearson’s chi-square test to compare discrete variables. For the comparison of continuous variables, Student’s t-test was used in normal distribution and Mann Whitney test in non-normal. The sensitivity, specificity and area under the ROC curve (AUC) were calculated using ROC curves. The Youden’s index was used to find cutoff point for the best combination of sensitivity and specificity. The sensitivity, specificity hazard ratios (HRs) and corresponding 95% confidence intervals (CIs) were calculated for predictors that were significant in the multivariate analysis. A two-sided *P* value < 0.05 was statistically significant. All statistical analyses were performed using SPSS for Windows software (ver. 20.0; IBM Corp., Armonk, NY, USA).

## Results

Overall, 729 COPD patients with severe exacerbations were admitted to hospital during this study. Of the 729 patients, 382 met exclusion criteria, thus 347 patients were finally included. The median age was 72.73 ± 9.38, and 73.2% (254/347) were male. Also, 28.8% (100/347) and 13.8% (48/347) of patients had blood eosinophilia during exacerbations based on the cut-off of ≥ 2% of total white cell counts and the cell counts (≥ 300 cells), respectively. Additionally, 30.5% (106/347) of patients had more than one hospitalization in a previous year due to COPD AE. Too, 34.3% (119/347) and 47.8% (166/347) were Global Initiative for Chronic Obstructive Lung Disease (GOLD) 2 and GOLD 3, respectively (Table 1).

**Table 1:** Baseline characteristics of COPD patients with acute exacerbations

	< 2% vs. ≥ 2% eosinophils			< 300 cells per µL vs. ≥ 300 cells per uL		
	Non-eosinophilic (n = 247)	Eosinophilic (n = 100)	*P* value	Non-eosinophilic (n = 299)	Eosinophilic (n = 48)	*P* value
Male	169 (68.4)	85 (85.0)	0.002	217 (72.6)	37 (77.1)	0.513
Age (year)	73.31 ± 8.83	71.30 ± 10.55	0.071	73.36 ± 8.99	68.79 ± 10.83	0.002
BMI (kg/m^2^)	21.68 ± 3.95	22.41 ± 3.51	0.134	21.82 ± 3.86	22.35 ± 3.66	0.409
Allergy history	4 (1.6)	7 (7.0)	0.032	5 (1.7)	6 (12.5)	< 0.001
Asthma history	52 (21.1)	17 (17.0)	0.392	60 (20.1)	9 (18.8)	0.832
Smoking history						
Never	75 (30.4)	17 (17.0)	0.011	82 (27.4)	10 (20.8)	0.337
Ex-smoker	110 (44.5)	56 (56.0)	0.053	143 (47.8)	23 (47.9)	0.991
Current smoker	52 (21.1)	22 (22.0)	0.845	62 (20.7)	12 (25.0)	0.503
Smoking (pack-year)	36.96 ± 32.23	44.60 ± 28.76	0.072	39.53 ± 32.72	37.43 ± 20.98	0.714
Blood eosinophil count at stable state	183.78 ± 150.37	284.33 ± 244.00	< 0.001	192.98 ± 153.57	335.98 ± 302.54	< 0.001
≥ 1 hospital admission in the previous year	81 (32.8)	25 (25.0)	0.153	95 (31.8)	11 (22.9)	0.216
COPD medication						
ICS	7 (2.8)	1 (1.0)	0.303	7 (2.3)	1 (2.1)	0.912
LAMA	123 (49.8)	44 (44.0)	0.328	144 (48.2)	23 (47.9)	0.975
LABA	12 (4.9)	6 (6.0)	0.664	15 (5.0)	3 (6.3)	0.721
ICS + LABA	129 (52.2)	32 (32.0)	0.001	146 (48.8)	15 (31.3)	0.023
PDE4 inhibitor	15 (6.1)	1 (1.0)	0.041	16 (5.4)	0 (0.0)	0.101
Gold						
1	15 (6.1)	6 (6.0)	0.979	18 (6.0)	3 (6.3)	0.951
2	81 (32.8)	38 (38.0)	0.355	101 (33.8)	18 (37.5)	0.614
3	118 (47.8)	48 (48.0)	0.969	143 (47.8)	23 (47.9)	0.991
4	33 (13.4)	8 (8.0)	0.161	37 (12.4)	4 (8.3)	0.421
Post-BD FEV_1_/FVC	45.28 ± 11.38	44.90 ± 10.76	0.774	45.07 ± 11.43	45.82 ± 9.68	0.669
Post-BD FVC (L)	2.38 ± 1.52	2.66 ± 0.85	0.081	2.43 ± 1.43	2.65 ± 0.83	0.307
Post-BD FVC (%)	73.65 ± 22.22	78.72 ± 24.49	0.063	74.54 ± 22.39	78.70 ± 26.31	0.244
Post-BD FEV_1_ (L)	1.01 ± 0.41	1.19 ± 0.47	0.001	1.01 ± 0.41	1.19 ± 0.47	0.001
Post-BD FEV_1_ (%)	47.86 ± 17.51	50.36 ± 20.86	0.255	47.86 ± 17.51	50.36 ± 20.86	0.255
Treatment during AE						
Steroid only	11 (4.5)	20 (20.0)	< 0.001	18 (6.0)	13 (27.1)	< 0.001
Antibiotics only	14 (5.7)	3 (3.0)	0.297	15 (5.0)	2 (4.2)	0.800
Steroid + antibiotics	216 (87.4)	68 (68.0)	< 0.001	253 (84.6)	31 (64.6)	0.001

Values are expressed as number (%) or mean ± SD

COPD, chronic obstructive pulmonary disease, BMI, body mass index, ICS, inhaled corticosteroids, LAMA, long acting muscarinic antagonist, LABA, long acting beta agonist, PDE4, phosphodiesterase-4, GOLD, global initiative for chronic obstructive lung disease, BD, bronchodilator, FEV_*1*_, forced expiratory volume in 1 s, FVC, forced vital capacity

**Fig. 1:**
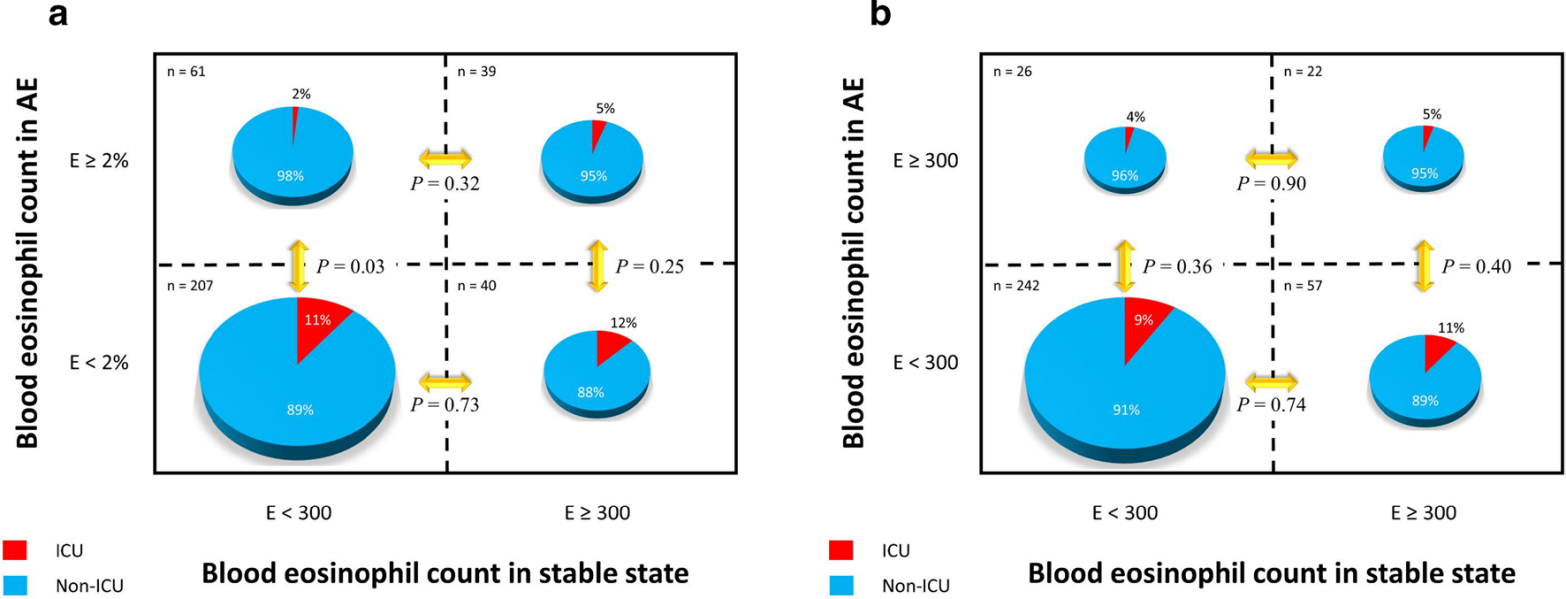
The difference of ICU admission rates among the four groups according to eosinophilia at the stable status and AE in COPD patients based on **a** eosinophil percentage cut off as 2% and **b** eosinophil count cut off as 300 cells/uL. ICU, intensive care unit; AE, acute exacerbations; COPD, chronic obstructive pulmonary disease

Compared to patients without eosinophilia, those with eosinophilia (defined as eosinophils ≥ 2%) had the lower rate of ICU admission (3.0% vs. 10.9%, *P* = 0.017). The overall mortality was not different between two groups, but there was a tendency to have higher early mortality in patients without eosinophilia (2.8% vs. 0.0%, *P* = 0.089). In patients with eosinophilia based on cell counts (≥ 300 cells), duration of MV (7.88 ± 9.27 vs. 10.91 ± 21.91 days, *P* = 0.004) was shorter compared to those without eosinophilia. The overall mortality was not different between the two groups (Table 2). In patients with non-eosinophilia based on cell counts (< 300 cells) in stable status, the cases with eosinophilia based on eosinophils ≥ 2% at AE had significant lower ICU admission (*P* = 0.03) than eosinophil < 2% at AE. However, ICU admission rates of other groups was not different (Fig. 1a, b).

**Table 2:** The clinical outcomes of COPD patients with eosinophilia

	< 2% vs. ≥ 2 eosinophils			< 300 cells per µL vs. ≥ 300 cells per uL		
	Non-eosinophilic (n = 247)	Eosinophilic (n = 100)	*P* value	Non-eosinophilic (n = 299)	Eosinophilic (n = 48)	*P* value
Length of hospital stay (days)	8.00 (6.00–13.00)	6.00 (4.00–10.00)	< 0.001	8.00 (6.00–12.00)	5.50 (3.25–9.50)	0.001
ICU admission	27 (10.9)	3 (3.0)	0.017	28 (9.4)	2 (4.2)	0.234
MV	21 (8.5)	3 (3.0)	0.067	22 (7.4)	2 (4.2)	0.419
Duration of MV (days)	5.50 (3.00–16.50)	10.00 (0.00–33.00)	0.829	5.00 (3.00–16.00)	37.00 (10.00 – 33.00)	0.142
Non-invasive ventilation	5 (2.0)	0 (0.0)	0.152	5 (1.7)	0 (0.0)	0.367
Treatment results						
Resolve	235 (95.1)	98 (98.0)	0.220	286 (95.7)	47 (97.9)	0.459
Mortality	12 (4.9)	2 (2.0)	0.220	13 (4.3)	1 (2.1)	0.459
Death within						
28 days	7 (2.8)	0 (0.0)	0.089	7 (2.3)	0 (0.0)	0.284
Death after						
28 days	5 (2.0)	2 (2.0)	0.988	6 (2.0)	1 (2.1)	0.972

Values are expressed as median [1st, 3rd quartile]

COPD, chronic obstructive pulmonary disease, ICU, intensive care unit, MV, mechanical ventilation

**Fig. 2:**
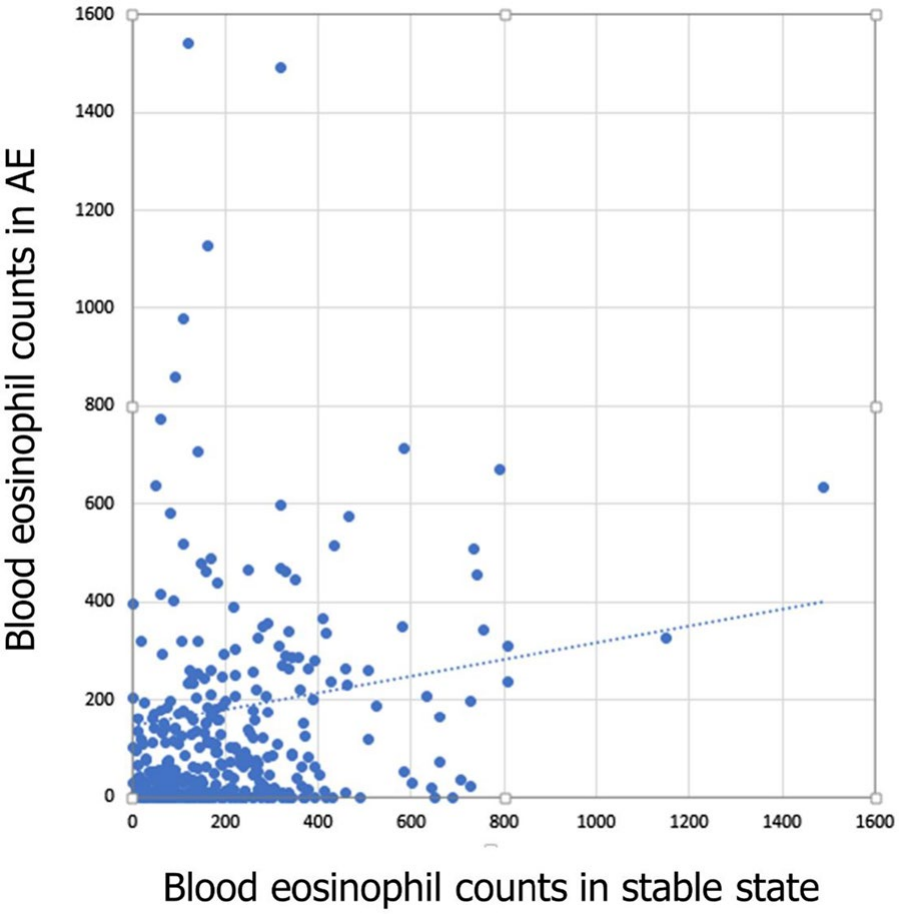
Scatterplot showing the correlation of eosinophil counts at stable COPD and acute exacerbations. COPD, chronic obstructive pulmonary disease; AE, acute exacerbations

We investigated the association between eosinophil counts in AE COPD and stable COPD. There was significant positive correlation of eosinophil counts between stable COPD and AE COPD (r = 0.156, *P* = 0.026) (Fig. 2).

**Fig. 3:**
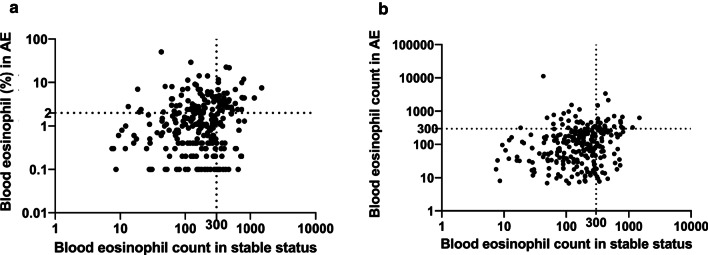
The distributions of eosinophil count at stable COPD and acute exacerbations based on (a) eosinophil percentage cut off as 2% and (b) eosinophil count cut off as 300 cells/uL. COPD, chronic obstructive pulmonary disease; AE, acute exacerbations

The mean blood eosinophil count was 182.06 ± 657.28/uL during COPD AE. The definition of eosinophilic exacerbation of COPD was based on two thresholds: ≥ 2% or ≥ 300 cells/µL, as defined by previous studies [11, 12]. The proportion of the concordant group of eosinophilia between stable status and COPD AE (eosinophil count ≥ 300 at stable status and eosinophil count ≥ 2% at AE or eosinophil count < 300 at stable status and eosinophil count < 2% at AE) was 70.9% (246/347) and that of the discordant group was 29.1% (101/347). When the definition of eosinophilia based on cell counts ≥ 300 in both conditions, the proportion of the concordant group (eosinophil counts ≥ 300 at stable status and eosinophil counts ≥ 300 at AE or eosinophil count < 300 at stable status and eosinophil counts < 300 at AE) was 76.1% (264/347) and that of the discordant group was 23.9% (83/347) (Fig. 3a, b).

**Fig. 4:**
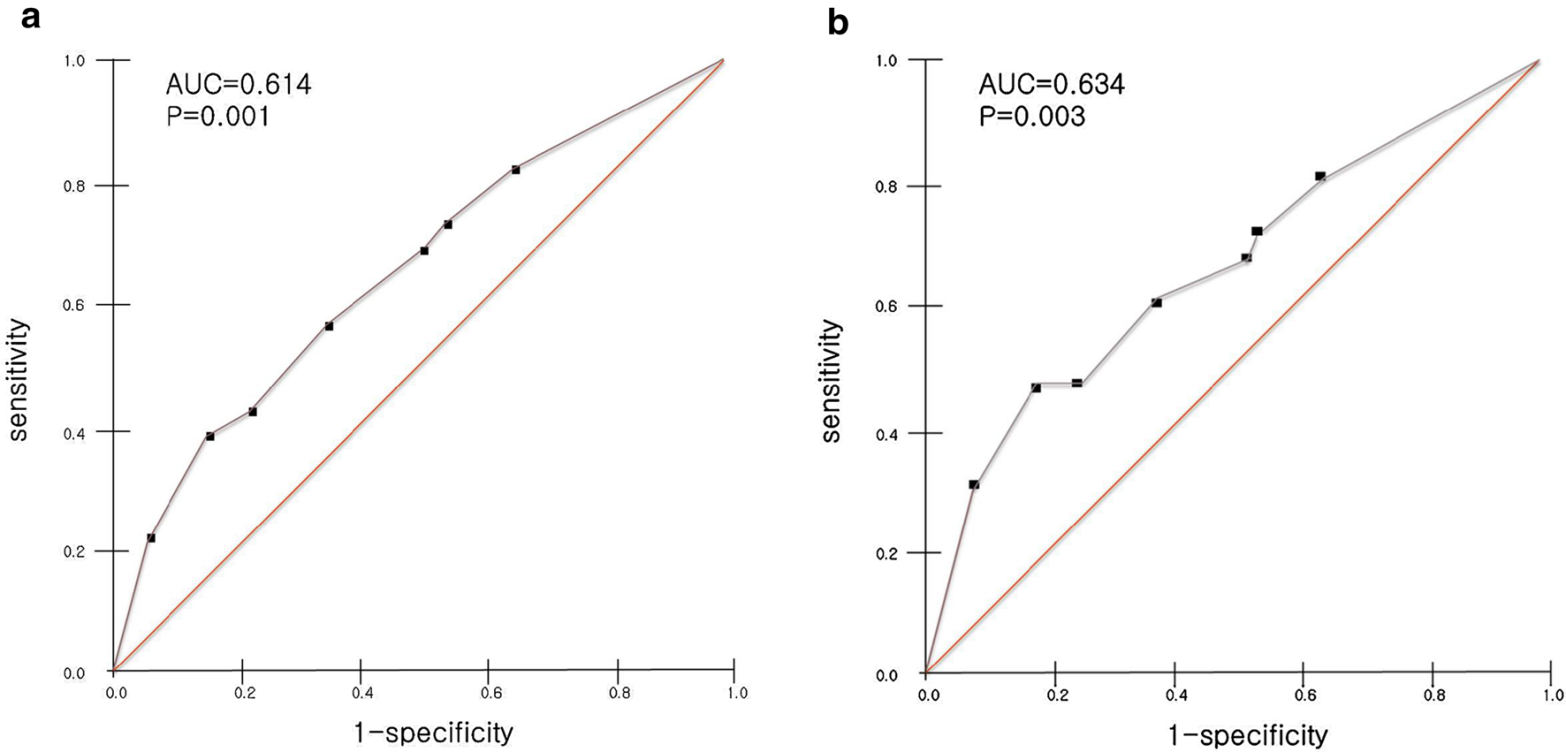
Receiver operating characteristic (ROC) curves for the absolute blood eosinophil count at the stable state to predict eosinophilic exacerbation based on **a** eosinophil percentage cut off as 2% and **b** eosinophil count cut off as 300 cells/uL

The best cut-off value of blood eosinophil count for the prediction of eosinophilic COPD exacerbations based on blood eosinophil count ≥ 2% was 300 cells/µL (area under the ROC curve (AUC) 0.614, *P* = 0.001, 39% sensitivity, 83.8% specificity). When the eosinophilic COPD exacerbation was based on the blood eosinophil count ≥ 300 cells/µL, the best cut-off value of blood eosinophil count for the prediction of eosinophilic COPD exacerbation was also 300 per uL (AUC 0.634, *P* = 0.046, 45.8% sensitivity, 80.9% specificity) (Table 3, Fig. 4, Additional file 1: Figure E1).

**Table 3:** Prediction of eosinophilic exacerbation by eosinophil levels stratified in stable COPD

Cut off	AUC	*P* value	Sensitivity	Specificity	PPV	NPV
Eosinophilic exacerbation (cut off eosinophil 2%)						
100	0.579	0.021	81	34.8	33.5	81.9
150	0.585	0.013	68	49.0	35.1	79.1
300	0.614	0.001	39	83.8	49.4	77.2
400	0.576	0.027	22	93.1	56.4	74.7
2%	0.587	0.011	72	45.3	34.8	80
3%	0.600	0.004	56	64	38.6	78.2
4%	0.599	0.004	42	77.7	43.3	76.8
Eosinophilic exacerbation (cut off eosinophil 300 cells/uL)						
100	0.567	0.042	81.3	32.1	16.1	91.4
150	0.562	0.044	66.7	45.8	16.5	89.5
300	0.634	0.046	45.8	80.9	27.8	90.3
400	0.616	0.048	31.3	92.0	38.5	89.3
2%	0.577	0.043	72.9	42.5	16.9	90.7
3%	0.608	0.044	60.4	61.2	20.0	90.6
4%	0.616	0.046	47.9	75.3	23.7	90

COPD, chronic obstructive pulmonary disease, AUC, area under the ROC curve, PPV, positive predictive value, NPV, negative predictive value

Eosinophilia (cut off eosinophil count 300 cells/uL) at stable COPD was independently associated with eosinophilic exacerbations (based on cut off eosinophil 2% or eosinophil count 300 cells/uL) after adjustment of age, gender, lung function, and medications including inhaled corticosteroid (ICS)s (Table 4).

**Table 4:** Association between eosinophilia at the stable state and eosinophilic exacerbations

Variables	OR	95% CI	*P* value
Eosinophilic exacerbations (cut off eosinophil 2%)			
Age	0.975	0.949–1.002	0.074
Male	2.542	1.347–4.799	0.004
Post BD FEV_1_ (%)	1.008	0.994–1.021	0.252
ICS containing inhaler	2.421	1.446–4.054	0.001
Eosinophilia at stable state (cut off eosihophil 300 cells/uL)	2.962	1.704–5.150	< 0.001
Eosinophilic exacerbations (cut off eosinophil count 300 cells/uL)			
Age	0.951	0.920–0.984	0.003
Male	1.113	0.524–2.366	1.113
Post BD FEV_1_ (%)	1.011	0.995–1.028	1.011
ICS containing inhaler	1.921	0.977–3.777	0.059
Eosinophilia at stable state (cut off eosihophil 300 cells/uL)	3.129	1.608–6.089	0.001

OR, odds ratio, CI, confidence interval, BD, bronchodilator, FEV_1,_ forced expiratory volume in 1 s, ICS, inhaled corticosteroid

## Discussion

In our study, COPD patients with eosinophilia during AE (defined as eosinophils ≥ 2%) had the lower rate of ICU admission. In patients with eosinophilic exacerbations based on cell counts (≥ 300 cells), duration of MV was shorter compared to those without eosinophilia. There was significant positive correlation of eosinophil counts between stable COPD and AECOPD. The best cut-off value of blood eosinophil count in stable status for the prediction of eosinophilic COPD exacerbations based on blood eosinophil count ≥ 2% was 300 cells/µL (AUC 0.614, *P* = 0.001, 39% sensitivity, 83.8% specificity). When the eosinophilic COPD exacerbation was based on the blood eosinophil count ≥ 300 cells/µL, the best cut-off value of blood eosinophil count in stable status for the prediction of eosinophilic COPD exacerbation was also 300 cells/uL (AUC 0.634, *P* = 0.046, 45.8% sensitivity, 80.9% specificity).

Bafadhel et al. stratified into eosinophilic exacerbations if the peripheral blood eosinophil on admission was ≥ 200 cells/µL and/or ≥ 2% of the total leukocyte count [1, 13]. Patients with severe eosinophilic exacerbation of COPD had a shorter stay [11]. In the use of an alternative cut-off level (eosinophil counts ≥ 300 cells/µL), patients with eosinophilia had higher frequency of readmission for AECOPD during one-year follow up [12]. In severe AECOPD requiring hospitalization, patients with eosinophilia showed prompt response to treatment with shorter hospital stay [11]. In our study, COPD patients with eosinophilic AE showed lower rate of ICU admission.

Blood eosinophilia at stable COPD is associated with higher exacerbation rates [14, 15]. Elevated blood eosinophil counts predict COPD exacerbation risk in ex-smokers [16]. Also, blood eosinophil count above 300 cells/uL increased risk of exacerbations in the COPDGene study [17]. Eosinophilic COPD is a distinct phenotype of the disease, and stable COPD and AE COPD with blood eosinophilia have significant clinical characteristics compared to non-eosinophilic patients[7, 18]. However, there few studies on the association between eosinophil counts in AECOPD and stable COPD and the thresholds of blood eosinophil count at stable COPD to predict eosinophilic AECOPD.

Raised blood eosinophil count (cut off 280) is common in COPD patients (about 40%) and suggested as a biomarker to predict the response of COPD patients to ICS. Siddiqui et al. reported clinical benefit from maintenance treatment with ICS in COPD when the blood eosinophil count was > 280/uL [19]. The 2020 GOLD document recommends a ICS therapy for initial treatment in patients with an eosinophil count greater than 300 cells/uL or those with history of, or concomitant, asthma. The threshold of a blood eosinophil count > 300 cells/uL is suggested as a biomarker to identify patients with the greatest likelihood of treatment benefit with ICS [20].

The association between eosinophilic inflammation of COPD, its dynamics and exacerbation risk are controversial. Schumann et al. suggested that blood eosinophil levels are variable throughout the course of COPD and phenotyping are difficult based on a single measurements [21]. In the ECLIPSE study, half of the patients were an intermittent group with variable eosinophil counts that oscillated above and below 2% [22]. However, Kim et al. reported that blood eosinophils at a time-point were a useful predictor of being in the persistent eosinophilia group over the next 12 months demonstrating longitudinal stability of blood eosinophilic inflammation within individuals [10].

In our study, there was significant positive correlation of eosinophil counts between stable COPD and AECOPD. Our finding is consistent with previous studies. In AERIS cohort, eosinophilic inflammation was more prevalent at exacerbation in patients with predominantly raised eosinophils at stable COPD [10]. Also, we demonstrated that the best cut-off value for the prediction of eosinophilic COPD exacerbation based on blood eosinophil count ≥ 2% or ≥ 300 cells/µL was blood eosinophil count ≥ 300 cells/µL in both cases. The thresholds of blood eosinophil counts to predict exacerbation risk, response to ICS and airway eosinophilia have been investigated, but those of specific blood eosinophil counts or percentage in stable state to predict eosinophilic exacerbations have not been investigated in COPD patients [14, 17, 19, 23].

This study has several limitations. First, this is a retrospective study, so our results may be confounded by unmeasured covariates. Second, patients with intermittent eosinophilia were not identified because identifying blood eosinophil count at all visits to outpatient clinic were not performed. In the ECLIPSE cohort study, the intermittent group comprised 49.0% of all subjects [22]. In our study, patients with intermittent eosinophilia could be included in the eosinophilic or non-eosinophilic group. Third, we included patients with severe AECOPD requiring hospital admission, so it is difficult to apply our results generally to other COPD populations such as moderate AECOPD. Fourth, we included 69 (19.9%) patients with history of asthma what could have confounded the results. ACO and COPD without asthma differ in terms of prognosis, treatment and clinical course. Finally, in some patients, blood eosinophils in stable state were evaluated after an exacerbation, what makes it difficult to draw conclusions on prediction.

## Conclusions

We demonstrated the association between blood eosinophil counts at stable COPD and those with AECOPD. Patients with AECOPD showed lower rate of ICU admission and shorter duration of MV during admission. The thresholds of blood counts at stable COPD to predict eosinophilic exacerbations was 300 cells/µL. Further and prospective studies in other population should validate our results.

## Supplementary Information

**Additional file 1: Figure E1**. Receiver operating characteristic for blood eosinophil (count & %) at exacerbation predicting sputum eosinophilia > 3% (n = 210) at exacerbation. At exacerbations blood eosinophils ≥ 2% cut point was 79.6% sensitive and 55.3% specific in identifying sputum eosinophils (> 3%)

## Data Availability

Data are available from the corresponding author upon a reasonable request.

## Abbreviations

COPD: Chronic obstructive pulmonary disease
AE: Acute exacerbations
ROC: Receiver operating characteristic
AUC: Area under the ROC curve
FEV_1_: Forced expiratory volume in 1s
FVC: Forced vital capacity
ICU: Intensive care unit
MV: Mechanical ventilation
HRs: Hazard ratios
CIs: Confidence intervals
GOLD: Global initiative for chronic obstructive lung disease
ICS: Inhaled corticosteroid
BMI: Body mass index
DM: Diabetes mellitus
MI: Myocardial infarction
CHF: Congestive heart failure
CVA: Cerebrovascular accident
PY: Pack-year
ER: Emergency room
LAMA: Long acting muscarinic antagonist
LABA: Long acting beta agonist
PDE4: Phosphodiesterase-4
PPV: Positive predictive value
NPV: Negative predictive value
OR: Odds ratio
BD: Bronchodilator

## References

[1] Bafadhel M, McKenna S, Terry S, Mistry V, Reid C, Haldar P, McCormick M, Haldar K, Kebadze T, Duvoix A (2011). Acute exacerbations of chronic obstructive pulmonary disease: identification of biologic clusters and their biomarkers. Am J Respir Crit Care Med.

[2] Bafadhel M, McKenna S, Terry S, Mistry V, Pancholi M, Venge P, Lomas DA, Barer MR, Johnston SL, Pavord ID (2012). Blood eosinophils to direct corticosteroid treatment of exacerbations of chronic obstructive pulmonary disease: a randomized placebo-controlled trial. Am J Respir Crit Care Med.

[3] Negewo NA, McDonald VM, Baines KJ, Wark PA, Simpson JL, Jones PW, Gibson PG (2016). Peripheral blood eosinophils: a surrogate marker for airway eosinophilia in stable COPD. Int J Chron Obstruct Pulmon Dis.

[4] MacDonald MI, Osadnik CR, Bulfin L, Hamza K, Leong P, Wong A, King PT, Bardin PG (2019). Low and high blood eosinophil counts as biomarkers in hospitalized acute exacerbations of COPD. Chest.

[5] Alaithan AM, Memon JI, Rehmani RS, Qureshi AA, Salam A (2012). Chronic obstructive pulmonary disease: hospital and intensive care unit outcomes in the Kingdom of Saudi Arabia. Int J Chron Obstruct Pulmon Dis.

[6] Kitaguchi Y, Komatsu Y, Fujimoto K, Hanaoka M, Kubo K (2012). Sputum eosinophilia can predict responsiveness to inhaled corticosteroid treatment in patients with overlap syndrome of COPD and asthma. Int J Chron Obstruct Pulmon Dis.

[7] Kang HS, Rhee CK, Kim SK, Kim JW, Lee SH, Yoon HK, Ahn JH, Kim YH (2016). Comparison of the clinical characteristics and treatment outcomes of patients requiring hospital admission to treat eosinophilic and neutrophilic exacerbations of COPD. Int J Chron Obstruct Pulmon Dis.

[8] Serafino-Agrusa L, Scichilone N, Spatafora M, Battaglia S (2016). Blood eosinophils and treatment response in hospitalized exacerbations of chronic obstructive pulmonary disease: a case-control study. Pulm Pharmacol Ther.

[9] Schumann DM, Tamm M, Kostikas K, Stolz D (2019). Stability of the blood eosinophilic phenotype in stable and exacerbated COPD. Chest.

[10] Kim VL, Coombs NA, Staples KJ, Ostridge KK, Williams NP, Wootton SA, Devaster JM, Aris E, Clarke SC, Tuck AC (2017). Impact and associations of eosinophilic inflammation in COPD: analysis of the AERIS cohort. Eur Respir J.

[11] Ryerson CJ, Cottin V, Brown KK, Collard HR (2015). Acute exacerbation of idiopathic pulmonary fibrosis: shifting the paradigm. Eur Respir J.

[12] Hasegawa K, Camargo CA (2016). Prevalence of blood eosinophilia in hospitalized patients with acute exacerbation of COPD. Respirology.

[13] Bafadhel M, Greening NJ, Harvey-Dunstan TC, Williams JE, Morgan MD, Brightling CE, Hussain SF, Pavord ID, Singh SJ, Steiner MC (2016). Blood eosinophils and outcomes in severe hospitalized exacerbations of COPD. Chest.

[14] Watz H, Tetzlaff K, Wouters EF, Kirsten A, Magnussen H, Rodriguez-Roisin R, Vogelmeier C, Fabbri LM, Chanez P, Dahl R (2016). Blood eosinophil count and exacerbations in severe chronic obstructive pulmonary disease after withdrawal of inhaled corticosteroids: a post-hoc analysis of the WISDOM trial. Lancet Respir Med.

[15] Vestbo J, Vogelmeier CF, Small M, Siddall J, Fogel R, Kostikas K (2019). Inhaled corticosteroid use by exacerbations and eosinophils: a real-world COPD population. Int J Chron Obstruct Pulmon Dis.

[16] Kerkhof M, Sonnappa S, Postma DS, Brusselle G, Agusti A, Anzueto A, Jones R, Papi A, Pavord I, Pizzichini E (2017). Blood eosinophil count and exacerbation risk in patients with COPD. Eur Respir J.

[17] Yun JH, Lamb A, Chase R, Singh D, Parker MM, Saferali A, Vestbo J, Tal-Singer R, Castaldi PJ, Silverman EK (2018). Blood eosinophil count thresholds and exacerbations in patients with chronic obstructive pulmonary disease. J Allergy Clin Immunol.

[18] Casanova C, Celli BR, de-Torres JP, Martinez-Gonzalez C, Cosio BG, Pinto-Plata V, de Lucas-Ramos P, Divo M, Fuster A, Peces-Barba G *et al*: Prevalence of persistent blood eosinophilia: relation to outcomes in patients with COPD. Eur Respir J 2017, 50(5). 10.1183/13993003.01162-2017 29167301

[19] Siddiqui SH, Guasconi A, Vestbo J, Jones P, Agusti A, Paggiaro P, Wedzicha JA, Singh D (2015). Blood eosinophils: a biomarker of response to extrafine beclomethasone/formoterol in chronic obstructive pulmonary disease. Am J Respir Crit Care Med.

[20] **2020 GOLD REPORTS**.

[21] Schumann DM, Tamm M, Kostikas K, Stolz D: Stability of the blood eosinophilic phenotype in stable and exacerbated COPD. Chest 2019. 10.1016/j.chest.2019.04.012 31047957

[22] Singh D, Kolsum U, Brightling CE, Locantore N, Agusti A, Tal-Singer R (2014). Investigators E: **Eosinophilic inflammation in COPD: prevalence and clinical characteristics**. Eur Respir J.

[23] Barnes NC, Sharma R, Lettis S, Calverley PM (2016). Blood eosinophils as a marker of response to inhaled corticosteroids in COPD. Eur Respir J.

