# Supplementary figures and images for: The association between eosinophilic exacerbation and eosinophilic levels in stable COPD
Source: BMC Pulm Med. 2021 Mar 2;21:74. doi: 10.1186/s12890-021-01443-4 (PMC7923497; doi:10.1186/s12890-021-01443-4)

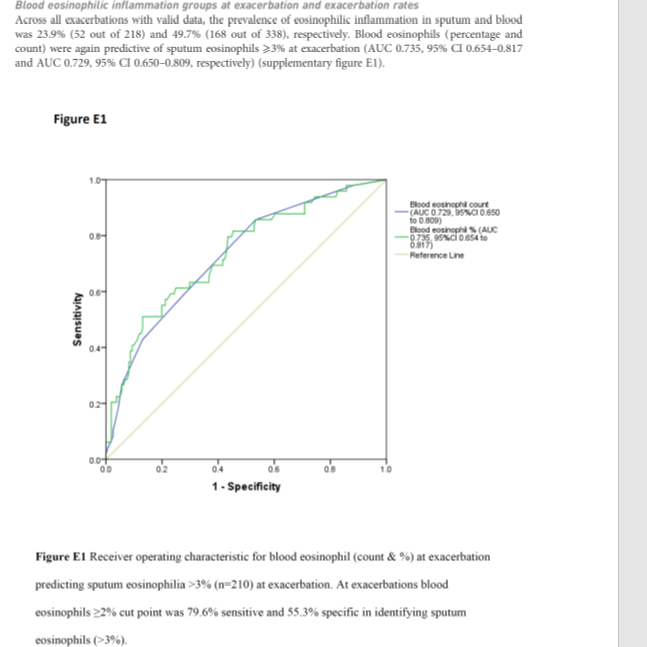

Supplement: Supplementary file 1 — Additional file 1: Figure E1. Receiver operating characteristic for blood eosinophil (count & %) at exacerbation predicting sputum eosinophilia > 3% (n = 210) at exacerbation. At exacerbations blood eosinophils ≥ 2% cut point was 79.6% sensitive and 55.3% specific in identifying sputum eosinophils (> 3%) [file 12890_2021_1443_MOESM1_ESM.png]
